# StackCirRNAPred: computational classification of long circRNA from other lncRNA based on stacking strategy

**DOI:** 10.1186/s12859-022-05118-7

**Published:** 2022-12-27

**Authors:** Xin Wang, Yadong Liu, Jie Li, Guohua Wang

**Affiliations:** School of Computer Science and Technology, Harbin Institute of Technology, Harbin, China

**Keywords:** Stacking strategy, circRNAs classification, Feature selection, Alu, Tandem repeats

## Abstract

**Background:**

CircRNAs are essential for the regulation of post-transcriptional gene expression, including as miRNA sponges, and play an important role in disease development. Some computational tools have been proposed recently to predict circRNA, since only one classifier is used, there is still much that can be done to improve the performance.

**Results:**

StackCirRNAPred was proposed, the computational classification of long circRNA from other lncRNA based on stacking strategy. In order to cope with the potential problem that a single feature might not be able to distinguish circRNA well from other lncRNA, we first extracted features from different sources, including nucleic acid composition, sequence spatial features and physicochemical properties, Alu and tandem repeats. We innovatively apply the stacking strategy to integrate the more advantageous classifiers of RF, LightGBM, XGBoost. This allows the model to incorporate these features more flexibly. StackCirRNAPred was found to be significantly better than other tools, with precision, accuracy, F1, recall and MCC of 0.843, 0.833, 0.831, 0.819 and 0.666 respectively. We tested it directly on the mouse dataset. StackCirRNAPred was still significantly better than other methods, with precision, accuracy, F1, recall and MCC of 0.837, 0.839, 0.839, 0.841, 0.677.

**Conclusions:**

We proposed StackCirRNAPred based on stacking strategy to distinguish long circRNAs from other lncRNAs. With the test results demonstrating the validity and robustness of StackCirRNAPred, we hope StackCirRNAPred will complement existing circRNA prediction methods and is helpful in down-stream research.

**Supplementary Information:**

The online version contains supplementary material available at 10.1186/s12859-022-05118-7.

## Background

Noncoding RNAs [1] (ncRNAs) are functional RNAs that are transcribed from DNA but cannot be translated into proteins. According to the length, ncRNA can be divided into short ncRNA (shorter than 200nt) and long non-coding RNA (lncRNA, more than 200nt). LncRNAs [2] are essential for the development and pathogenesis of disease as well as the control of genes. CircRNAs are closed-loop RNA molecules that participate in a variety of molecular functions of the transcriptional regulation [3] and translation into protein products [4].

CircRNAs were first discovered in plant viruses in the 1990s [5]. The early development in this subject may be rather gradual because linear RNAs predominate and circRNAs were previously thought to be a by-product of RNA splicing [6, 7]. With the development of biotechnology, circRNA detection tools have been developed one after another. According to the form of implementation, circRNA detection methods can be divided into three categories, including machine learning-based, back-splicing junction (BSJ)-based and integration-based.

BSJ-based circRNA detection method that identify circRNAs by identifying BSJ reads. Gao et al. [8] proposed CIRI2, a multithreaded recognition method using adaptive maximum likelihood. Smid et al. [9] proposed a splicing data-independent circRNA identification method to analyze the function of circRNAs in breast cancer. However, these methods have the disadvantage of high false positives, different algorithm implementations between different tools, and large differences in prediction results. The above problems are alleviated by the prediction methods based on multi-tool integration. CirComPara [10] is an automated pipeline for detection and annotation of circRNAs in RNA-Seq data. It integrates testrealign [11], CIRCexplorer [12], CIRI [13] and find_circ [14] four different back-splicing identification methods. CircRNAwrap [15] is a more comprehensive pipeline tool for detection and abundance quantification of circRNAs, using many techniques (find_circ, KNIFE [16], MapSplice [17], CIRI, CIRCexplorer, DCC [18], ACFS [19] and circRNA_finder [20]) in parallel for back splicing identification and construction of whole transcripts. But these tools have certain limitations, and most require RNA-Seq datasets as input. The development of machine learning techniques addresses this deficiency, and machine learning algorithms allow models to learn features directly from sequences. In 2015, Pan et al. [21] proposed the PredcircRNA calculation method, which uses a multicore learning algorithm to extract features from transcript sequences to predict circRNAs. WebCircRNA is a tool for predicting specific circRNAs in stem cells by using sequence features as input by a random forest model [22]. Niu et al. [23] at 2020 developed a new classifier, CirRNAPL, which uses the particle swarm optimization algorithm to adjust the extreme learning machine (ELM), extracts the computational composition of sequences, and predicts circRNAs by structural features. The extreme learning machine (ELM) [24] is an artificial neural network model with good generalization performance and learning ability. ELM only needs to set the structure of the network and no other parameters, so it has the features of simplicity and ease of use. The algorithm does not require additional adjustments during execution because the weights from the input layer to the hidden layer are chosen randomly all at once. Strong generalization ability and fast learning speed are its outstanding advantages. However, these tools use a single classifier, and there is still much that can be done to improve the performance.

Other sequences have been predicted using ensemble learning [25]. In this study, we focused on classifying long circRNAs from other lncRNAs and proposed StackCirRNAPred based on the stacking strategy. In order to cope with the potential problem that a single feature might not be able to distinguish circRNA well from other lncRNA, we first extracted features from different sources, including sequence k-mer composition, dinucleotide-based auto-cross covariance (DACC), open reading frame (ORF), series correlation pseudo dinucleotide composition (SCPseDNC), Alu, tandem repeats. To remove redundant features, the mRMR algorithm was used to select the best feature dataset. In the selection of classifiers, considering the heterogeneity of these features, combining different features and selecting a suitable classifier is a means to improve the recognition sensitivity and specificity. We innovatively apply the stacking strategy to integrate multiple more advantageous classifiers, which can predict circRNAs from multiple aspects, fuse these features more flexibly and improve model accuracy. To confirm the validity of our model, it was directly tested on mouse datasets and achieved good performance, indicating that the method has good generalization.

## Materials and methods

**Fig. 1:**
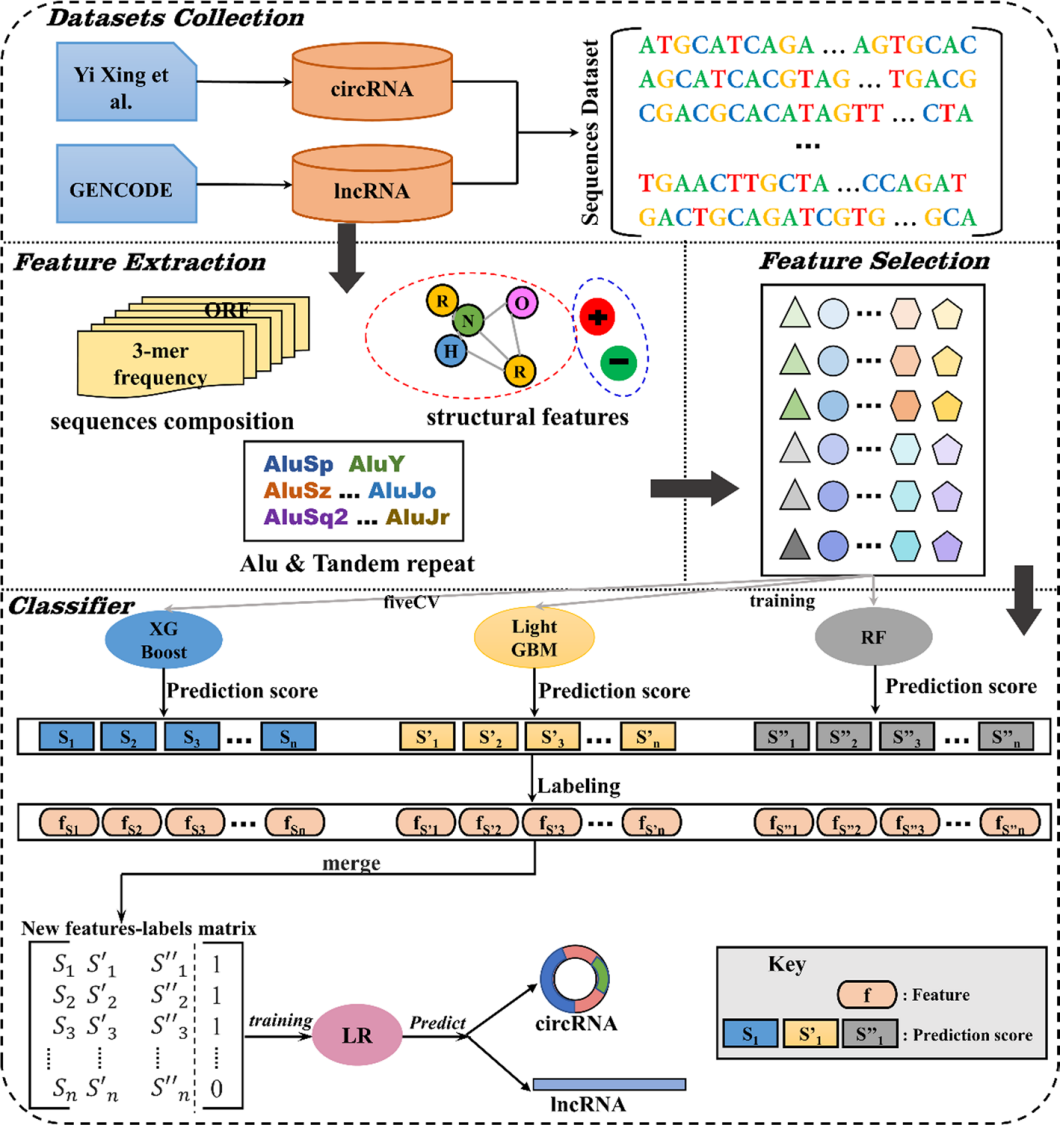
The overall workflow of StackCirRNAPred

StackCirRNAPred primarily consists of four parts (Fig. 1): (i) datasets collection, (ii) feature extraction: including nucleic acid composition, sequence spatial features and physicochemical properties, Alu and tandem repeats, (iii) feature selection and (iv) classifier.

### Dataset construction

In this study, Human (GRCh37) and mouse (NCBI37) reference genome files were downloaded from UCSC Genome Browser. Xing et al. [26] proposed isoCirc, the long-read sequencing method to reliably detect full-length circRNA isoforms using the experimental methods of negative enrichment (line RNA removal) and rolling circle amplification followed by Oxford nanopore long-read sequencing. Through the analysis of twelve human tissues and one human cell line, the investigators provided circRNA data, which not only included the circRNA data characterized by isoCirc, but also included in the circBase database. circBase [27] is a database of a large collection of other published experimentally validated circRNA transcripts. It organized and annotated circRNAs based on information from 9 published large-scale circRNA identification studies. We downloaded the circRNA annotation data from the paper of Xing et al. [28] We collected lncRNAs from GENCODE (GRCh37).

For circRNAs, we removed transcripts shorter than 200nt. For lncRNAs, we constructed a negative dataset consisting of other lncRNAs defined in GENCODE, such as sense overlapping, antisense, sense intronic, processed transcripts and lincRNAs [29]. To get rid of the redundancy and avoid bias, the CH-HIT software [30] was utilized by setting its cutoff threshold to 0.8. Finally, we obtained 39,260 circRNAs and 19,006 lncRNAs as the benchmark dataset.

To further expand the independent dataset to validate the model, we downloaded the mouse circRNA annotation bed file from circBase [27] and the lncRNAs sequence from GENCODE (Release M1). Through the same processing method as human, 1903 circRNA sequences and 5627 lncRNA sequences were used as another independent test set. All the above datasets can be obtained from an additional file (see Additional file 1).

### Feature extraction

We extracted 170 features, which are briefly described in Table 1.

**Table 1:** Extracted features list

Feature group	Feature names
Based on k-mer	64 trinucleotide frequencies
Based on open reading frame	ORF length, ORF coverage, ORF average coverage, ORF difference
Based on structural features	Dinucleotide-based Auto-Cross Covariance (DACC)
Based on physicochemical properties	Series correlation pseudo dinucleotide composition (SCPseDNC)
Based on repeats	Alu, tandem repeat

#### K-mer

The DNA or RNA sequences can be represented by the frequency of occurrence of $k$ adjacent nucleotides. Trinucleotide frequency has been used for circRNAs prediction. The Kmer ($k = 3$) descriptor can be defined as:

1 $$f\left( t \right) = \frac{N\left( t \right)}{N}, t \in \left\{ {AAA, AAC, AAG, \ldots , TTT } \right\}$$

$N$ represents the length of a nucleotide sequence and $N\left( t \right)$ represents the count of 3-mer type $t$.

#### ORF

Four features of ORF were extracted, including ORF length, ORF average coverage, ORF coverage and ORF difference. The putative ORF for each transcript sequence is the longest feasible open reading frame among the three reading frames.

1. ORF length has been reported useful for circRNA classification [21].
2. ORF coverage is the putative open reading frame divided by the length of the transcript.
3. ORF average coverage is the average length of the three open reading frames divided by the length of transcript.
4. ORF difference indicates the characteristic differences of the three ORFs. It is defined as:
  2 $$d = \frac{{\left( {x_{0} - x_{1} } \right)^{2} + \left( {x_{0} - x_{2} } \right)^{2} + \left( {x_{1} - x_{2} } \right)^{2} }}{2}$$

where $x_{0}$, $x_{1}$ and $x_{2}$ are the corresponding eigenvalues of the ORF sequences in the three reading frames.

#### Dinucleotide-based auto-cross covariance (DACC)

One of the six different kinds of autocorrelation encodings is DACC. By calculating the correlation between two properties, autocorrelation encoding [31] can convert nucleotide sequences of different lengths into a fixed-length vector. The DACC is a fusion of dinucleotide-based cross covariance (DCC) encoding and dinucleotide-based auto covariance (DAC). Six properties were used to calculate the DACC. Tilt, roll and twist reflect the changes in the up-and-down, front-to-back, and left–right angles of adjacent base space plane, respectively; rise, slide and shift reflect the changes in the distance between the up-and-down, front-to-back, and left–right relative positions of adjacent bases [32, 33]. These six properties allow us to deeply study the local conformational differences in sequences by quantitatively describing the changes in sequence spatial structure.

DAC calculates the correlation of identical physicochemical indices between two dinucleotides that are separated along the sequence along the lagging distance. The formula for DAC is:

3 $$DAC\left( {u, lag} \right) = \mathop \sum \limits_{i = 1}^{L - lag - 1} \left( {\left( {P_{u} \left( {R_{i} R_{i + 1} } \right) - \overline{{P_{u} }} } \right)\left( {P_{u} \left( {R_{i + lag} R_{i + lag + 1} } \right) - \overline{{P_{u} }} } \right)/\left( {L - lag - 1} \right)} \right)$$

where $u$ is a physicochemical index, $lag$ is a distance that separate two dinucleotide, $L$ represents the length of the nucleotide sequence, $P_{u} \left( {R_{i} R_{i + 1} } \right)$ is a numerical representation of the physicochemical property $u$ for the dinucleotide $R_{i} R_{i + 1}$ at position $i$, $\overline{{P_{u} }}$ is the average value for the physicochemical property $u$ throughout the entire sequence:

4 $$\overline{{P_{u} }} = \mathop \sum \limits_{j = 1}^{L - 1} P_{u} \left( {R_{J} R_{j + 1} } \right)/\left( {L - 1} \right)$$

The DAC vector has a dimension of $N \times LAG$, where $N$ represents the total of physicochemical properties and LAG denotes the greatest amount of $lag \left( {lag = 1, 2, \ldots , LAG} \right)$.

The DCC encoding is calculated as:

5 $$DCC\left( {u_{1} ,u_{2} , lag} \right) = \mathop \sum \limits_{i = 1}^{L - lag - 1} \left( {\left( {P_{{u_{1} }} \left( {R_{i} R_{i + 1} } \right) - \overline{P}_{{u_{1} }} } \right)\left( {P_{{u_{2} }} \left( {R_{i + lag} R_{i + lag + 1} } \right) - \overline{P}_{{u_{2} }} } \right)/\left( {L - lag - 1} \right)} \right)$$

$P_{{u_{a} }} \left( {R_{i} R_{i + 1} } \right)$ is a numerical representation of the physicochemical property $u_{a}$ for the dinucleotide $R_{i} R_{i + 1}$ at position $i$, and where $u_{1}$ and $u_{2}$ are separate physicochemical properties. The average value for the physicochemical property $u_{a}$ along the whole sequence is $\overline{P}_{{u_{a} }}$:

6 $$\overline{P}_{{u_{a} }} = \mathop \sum \limits_{j = 1}^{L - 1} P_{{u_{a} }} \left( {R_{J} R_{j + 1} } \right)/\left( {L - 1} \right)$$

The DCC vector has a dimension of $N \times \left( {N - 1} \right) \times LAG$, where $N$ represents the total of physicochemical properties and LAG is the highest value of $lag \left( {lag = 1, 2, \ldots , LAG} \right)$.

Thus, the dimension of the DACC encoding is $N \times N \times LAG$, where $N$ is the total number of physicochemical indices and $LAG$ is the maximum of the $lag$ ($lag = 1, 2, \ldots , LAG$).

#### Series correlation pseudo dinucleotide composition (SCPseDNC)

The Series Correlation Pseudo Dinucleotide Composition encoding [34] defines as:

7 $$D = \left[ {d_{1} ,d_{2} , \ldots ,d_{16} ,d_{16 + 1} , \ldots ,d_{16 + \lambda } ,d_{16 + \lambda + 1} , \ldots , d_{16 + \lambda \Lambda } } \right]^{T}$$

where:

8 $$d_{k} = \left\{ {\begin{array}{*{20}l} {\frac{{f_{k} }}{{\mathop \sum \nolimits_{i = 1}^{16} f_{i} + w\mathop \sum \nolimits_{j = 1}^{\lambda } \theta_{j} }},} \hfill & {\left( {1 \le k \le 16} \right)} \hfill \\ {\frac{{w\theta_{k - 16} }}{{\mathop \sum \nolimits_{i = 1}^{16} f_{i} + w\mathop \sum \nolimits_{j = 1}^{\lambda \Lambda } \theta_{j} }},} \hfill & {\left( {17 \le k \le 16 + \lambda \Lambda } \right)} \hfill \\ \end{array} } \right.$$

where $w$ represents the weight parameter in the range of 0 to 1, $f_{k}$ (k = 1, 2, …, 16) represents the frequency of dinucleotides in the sequence, $\lambda$ represents the correlation tier of the nucleotide sequence, $\theta_{j} \left( {j = 1,2, \ldots , \lambda } \right)$ represents the j-tier correlation factor is calculated:

9 $$\left\{ {\begin{array}{*{20}c} {\theta_{1} = \frac{1}{L - 3}\mathop \sum \limits_{i = 1}^{L - 3} J_{i,i + 1}^{1} } \\ \ldots \\ {\theta_{\Lambda } = \frac{1}{L - 3}\mathop \sum \limits_{i = 1}^{L - 3} J_{i,i + 1}^{\Lambda } } \\ \ldots \\ {\theta_{\lambda \Lambda } = \frac{1}{L - \lambda - 2}\mathop \sum \limits_{i = 1}^{L - \lambda - 2} J_{i,i + \lambda }^{\Lambda } } \\ \end{array} \left( {\lambda < L - 2} \right)} \right.$$

where the correlation function is calculated as:

10 $$\left\{ {\begin{array}{*{20}l} {J_{i,i + m}^{\xi } = P_{u} \left( {R_{i} R_{i + 1} } \right)P_{u} \left( {R_{i + m} R_{i + m + 1} } \right)} \hfill \\ {\xi = 1,2, \ldots ,\Lambda ; m = 1,2, \ldots ,\lambda ; i = 1,2, \ldots ,L - \lambda - 2} \hfill \\ \end{array} } \right.$$

where ${\Lambda }$ represents the count of physicochemical properties. Six DNA physicochemical metrics were utilized, including three distance variables (Shift, Slide, Rise) and three angle variables (Twist, Tilt, Roll).

#### Alu and tandem repeat

Studies have shown that the flanking introns of circRNA have Alu repeat enrichment, which is related to the biogenesis of some circRNAs [12]. We used the Table Browser tool in the UCSC Genome Browser to download the Alu bed annotation file from the RepeatMasker track. Therefore, we examined the two windows (1000nt and 2000nt) of the genome sequence that flank the reverse splicing site for each circRNA [26]. We count the number of Alu repeats for each window. CircRNAs are formed by exon head-to-tail splicing, and tandem repeats can significantly promote reverse splicing within genes. Thus, this study used Tandem Repeats Finder to extract the tandem repeat frequency in the sequences.

### Feature selection

Generally, as the feature dimension increases, it will lead to the following three problems, first, the disadvantage of overfitting is that the predictor has severe bias and extremely low generalization ability; second, information redundancy or noise will lead to misstatements error, resulting in poor prediction accuracy; in the end, unnecessary computation time will be added. Therefore, selecting the most helpful subset of features from a high-dimensional feature dataset is an important process to reduce noise, improve identification accuracy, avoid overfitting, and build robust models [35]. In this study, we used feature selection techniques to optimize the included features. Doing so not only provides a deeper understanding of intrinsic properties of circRNA sequences, but also provides the comprehensibility, scalability and accuracy of prediction models. Max-relevance and min-redundancy (mRMR) is a filtered feature selection method [36]. Its main goal is to minimizing the relevance between features while maximizing the relevance between features and categorical variables.

There is a key in mRMR called mutual information. In this study, the mutual information of two random variables X and Y is calculated as:

11 $$I\left( {X;Y} \right) = \mathop \sum \limits_{x\smallint X} \mathop \sum \limits_{y\smallint Y} p\left( {x,y} \right)\log \frac{{p\left( {x,y} \right)}}{p\left( x \right)p\left( y \right)}$$

The goal of the mRMR algorithm is to find a feature subset S that contains m{$x_{i}$} features. First find the maximum relevance of m features and category c, which is defined as:

12 $$\max D\left( {S,c} \right),D = \frac{1}{\left| S \right|}\mathop \sum \limits_{{x_{i} \smallint S}} I\left( {x_{i} ;c} \right)$$

where $x_{i}$ is the ith feature, c is a categorical variable, S is a feature subset.

The next step is to eliminate the redundancy between m features:

13 $$\min R\left( S \right),R = \frac{1}{{\left| S \right|^{2} }}\mathop \sum \limits_{{x_{i} ,x_{j} \smallint S}} I\left( {x_{i} ;x_{j} } \right)$$

Then integrate maximum relevance and minimum redundancy:

14 $$\max \Phi \left( {{\text{D}},{\text{R}}} \right),\Phi = {\text{D}} - {\text{R}}\;{\text{or}}\;\max \Phi \left( {{\text{D}},{\text{R}}} \right),\Phi = {\text{D}}/{\text{R}}$$

Finally, the feature set S with the maximum relevance and the minimum redundancy is obtained.

### Stacking strategy

Ensemble learning is to combine multiple single classifiers together to form a classifier with better generalization ability. Stacking is a hierarchical model integration strategy, that is, integrating multiple classifiers through one classifier [37]. The basic idea is to use the original dataset to train the first-layer classifiers, then use the classifiers to make predictions on the test dataset, and use the output values as the input values for training the second-layer classifier, and the original labels are used as the labels for the training data of the second layer, and the output values of the second layer are used as the final prediction results (see Fig. 1). Among them, the first-layer classifiers are called base learners, and the second-layer classifier for combination is called a meta-learner.

#### Base learners

Extreme gradient boosting (XGBoost) [38, 39] is a decision tree-based integrated machine learning algorithm that excels at performing predictions, processing missing values, and parallel computing. LightGBM is a boosting ensemble model developed by Microsoft, which supports parallel learning, can handle large-scale data, low memory usage, and has better accuracy [40]. Without feature selection, random forest can analyze any type of data with high accuracy and strong resistance to overfitting [41]. Therefore, in this study, we use XGBoost, LightGBM and RF as the base learner for the first layer.

**Fig. 2:**
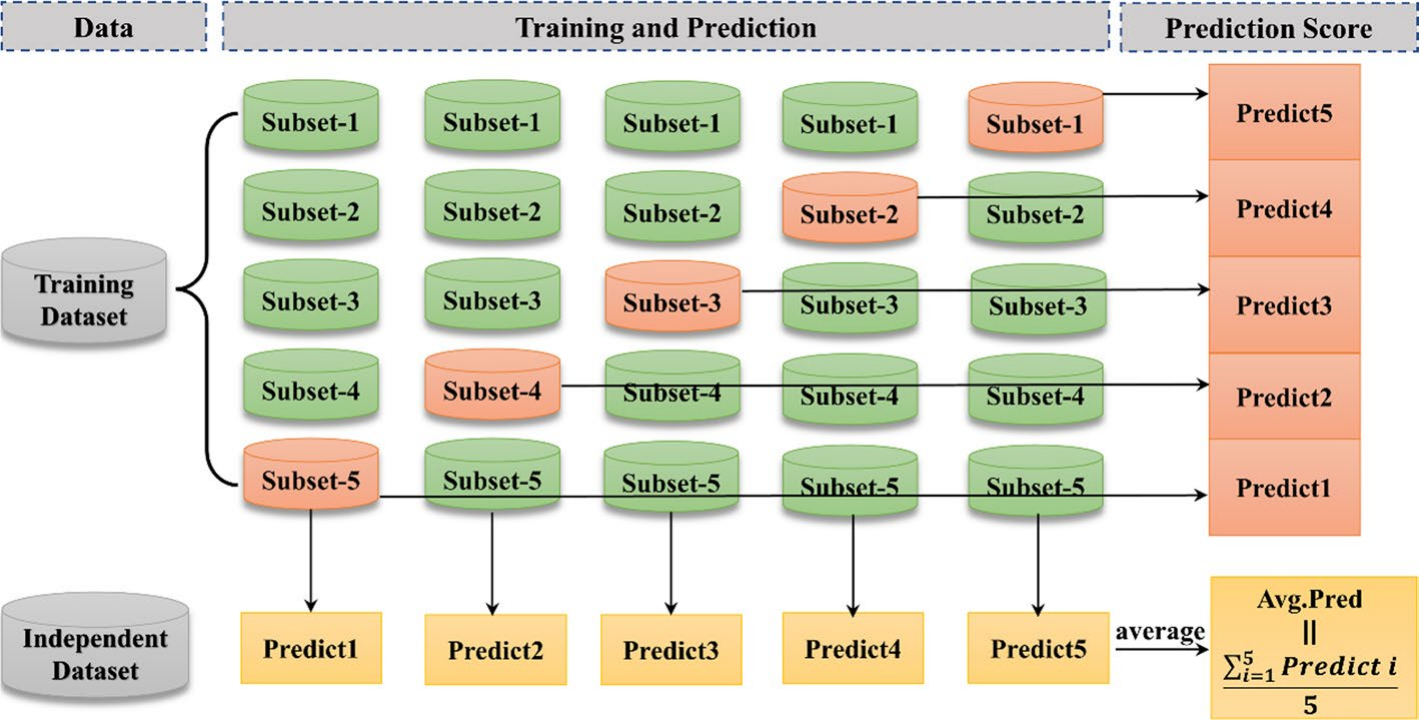
Five-fold cross-validation for each classifier in the first layer

In the training process, if LR is trained directly using the training set of the first layer learner, it will lead to the risk of overfitting. We performed five-fold cross-validation on each classifier for the first layer to prevent overfitting. See in Fig. 2, for each base learner, the training data set is divided into 5 equal-sized subsets, one is set aside as the validation dataset each time, while the other four are utilized to train the model. The trained model is used for predictions on the test dataset. It is performed five times so that each training sequence can get a prediction score. The prediction score of the base learning model on the test dataset is calculated by averaging the prediction scores of the five models. This will be the input to the second layer meta classifier.

#### Meta learner-logistic regression model

The logistic regression (LR) is a learning model commonly used to solve dichotomous classification problems [42]. LR classification has the benefits of small computational complexity, fast speed, little storage resources, and parallelism. It has been frequently utilized to address issues in the field of bioinformatics [43–45]. Using LR as a meta-classifier, the base learners from the first layer are integrated into the second layer in this study. The input data of the logistic regression classifier are the output probabilities of the first-layer primary learner, the labels of the raw data set are still the labels of the LR training dataset.

### Performance evaluation metrics

We employed standard performance metrics including accuracy (ACC), precision, recall, F1 value, specificity (Sp), and MCC. These metrics are defined as follows:

15 $$ACC = \frac{TP + TN}{{TP + FN + FP + TN}}$$

16 $$Precision = \frac{TP}{{TP + FP}}$$

17 $$Recall = \frac{TP}{{TP + FN}}$$

18 $$F_{1} = 2 \times \frac{TP}{{2TP + FP + FN}}$$

19 $$MCC = \frac{TP \times TN - FP \times FN}{{\sqrt {\left( {TP + FP} \right)\left( {TP + FN} \right)\left( {TN + FP} \right)\left( {TN + FN} \right)} }}$$

where TP (true positive), TN (true negative), FP (false positive) and FN (false negative).

## Results

### Feature optimization

**Fig. 3:**
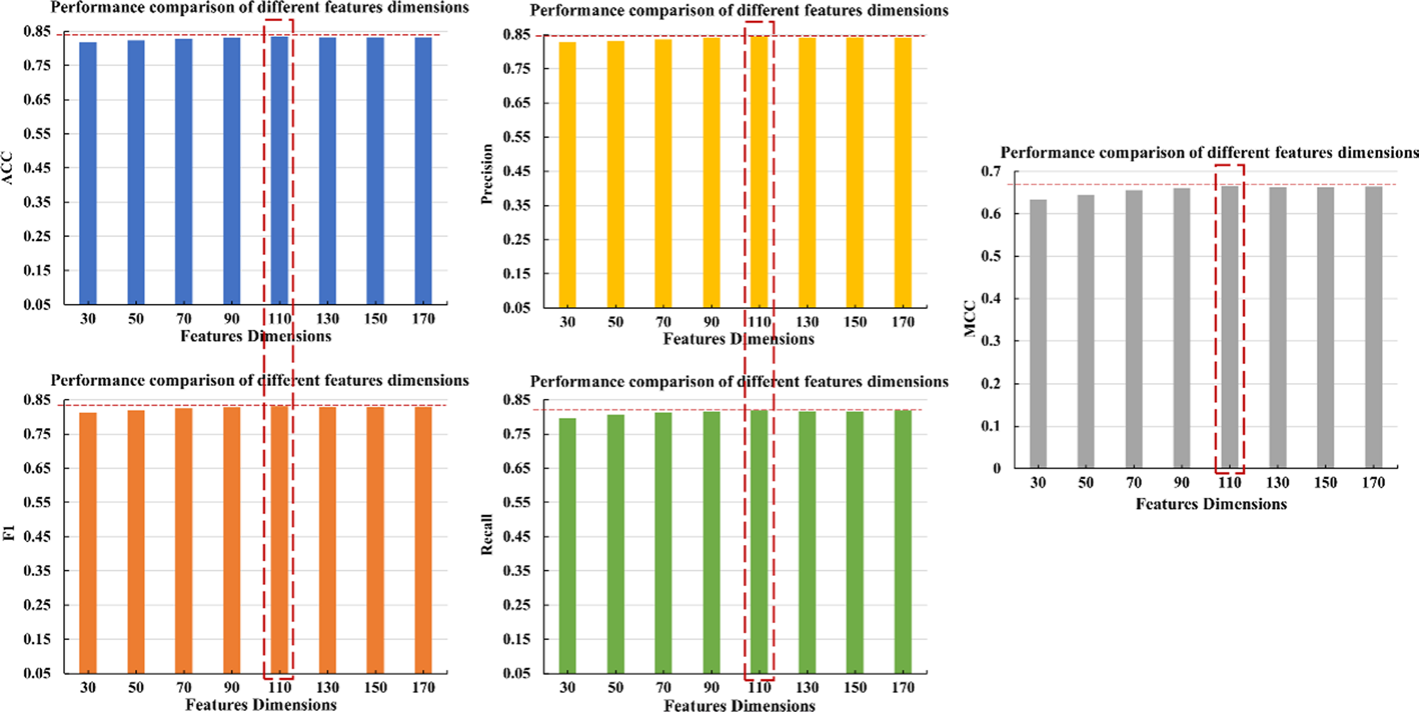
Comparison results of model performance metrics under different feature dimensions. When the feature dimension is 110, the performance evaluation metrics are all better than other feature dimensions

From the description in the feature extraction section above, we can see that we have extracted a total of 170-dimensional features. When performing feature selection, we set 8 feature selection dimensions of 30, 50, 70, 90, 110,130, 150,170. Models are trained on different feature datasets and then evaluated in a test data sets. See Fig. 3 for the results. When the feature dimension is 110, the five performance evaluation metrics of accuracy, precision, recall, F1 and MCC are all better than other feature dimensions. Therefore, in this study we used 110-dimensional features as the final feature dataset (see Additional file 2: Table S1).

### Comparison with base learners

We compared StackCirRNAPred with three base learners, XGBoost, LightGBM and RF, and see Table 2 for the results, with the optimal performance for each metric shown in bold. StackCirRNAPred outperformed all three base learners in all five metrics: ACC, precision, recall, F1 and MCC. This shows that StackCirRNAPred, constructed by fusing the three base learners XGBoost, LightGBM, and RF together through the stacking strategy, achieved better performance than each individual model and better integration of features from different sources.

**Table 2:** Comparison of the performance of StackCirRNAPred with each individual model

Model	ACC (%)	Precision (%)	Recall (%)	F1 (%)	MCC (%)
XGBoost	81.62	83.20	79.24	81.17	63.31
LightGBM	81.01	82.08	79.34	80.68	62.05
RF	80.54	81.33	79.27	80.29	61.51
StackCirRNAPred	**83.31**	**84.27**	**81.91**	**83.07**	**66.63**

Bold indicates the best performance for each metric

### Comparison with other tools on human dataset

**Fig. 4:**
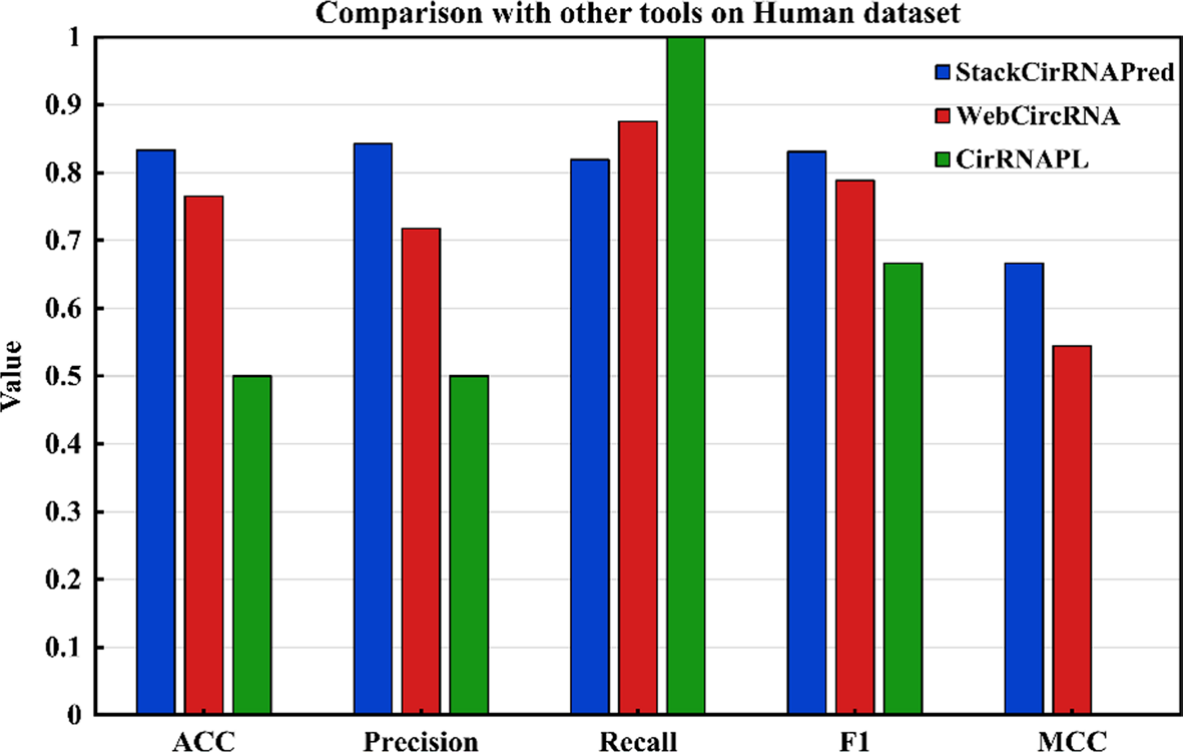
Comparison results with other tools on human dataset

We compared our method with other tools available to us. PredcircRNA uses a multi-core learning algorithm [21], and extract sequence features such as graph features and conservation scores to classify circRNAs and lncRNAs. PredicircRNATool [46] distinguishes circRNAs based on the SVM model by extracting flanking introns and thermodynamic dinucleotide properties as features. WebCircRNA is based on random forests and uses sequence-derived features to predict circRNA in stem cells [22]. CirRNAPL is a recently proposed circRNA prediction tool, which uses the particle swarm optimization algorithm to adjust the extreme learner [23]. It extracts the computational composition and structural features of the sequence. We cannot compare our method with theirs because they do not provide a web server or they are no longer available. So here we compare our method with WebCircRNA, CirRNAPL two tools. As shown in Fig. 4, WebCircRNA got the ACC, precision, recall, F1 and MCC with 0.765, 0.717, 0.875, 0.788 and 0.544. And it can be clearly found that the ACC, precision, F1 and MCC of StackCirRNAPred are significantly better than the other two tools, reaching 0.833, 0.843, 0.819, 0.831 and 0.666, respectively. In terms of recall, StackCirRNAPred is lower than the other two tools, but the web server provided by CirRNAPL has an excellent capacity to predict positive samples, it is found that the ability to identify negative samples is extremely weak and false positives are extremely high, so this is unstable. It is found that the overall effectiveness of StackCirRNAPred is the best.

### Comparison with other tools on mouse dataset

**Fig. 5:**
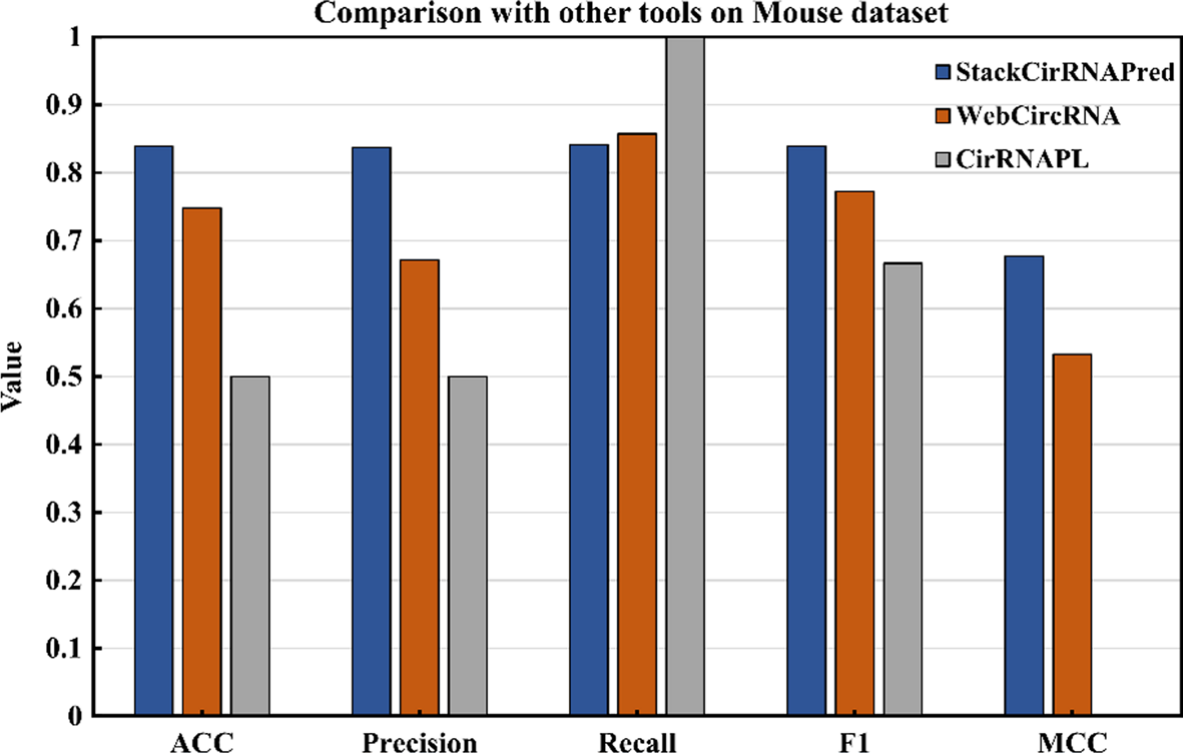
Comparison results with other tools on mouse dataset

To confirm the validity of our model, StackCirRNAPred and other two tools were directly tested and compared on mouse datasets. The results are shown in Fig. 5. For ACC, StackCirRNAPred better than WebCircRNA and CirRNAPL with 0.839, 0.748, 0.5. For precision and F1, StackCirRNAPred, WebCircRNA and CirRNAPL were 0.837/0.839, 0.671/0.771 and 0.5/0.667, respectively. CirRNAPL was also unstable that the ability to identify negative samples is extremely weak and false positives are extremely high. So, the recall of CirRNAPL cannot be calculated. The recall difference between StackCirRNAPred and WebCircRNA was very small with 0.841, 0.856. Therefore, even on mouse dataset, the identification performance is still better than other methods, which also shows the effectiveness and robustness of StackCirRNAPred.

## Discussion

CircRNAs belong to a subcategory of lncRNAs. With the development of sequencing technology, more and more circRNAs are annotated in the transcriptome. Unfortunately, distinguishing circRNAs from traditionally labeled lncRNAs remains a challenging problem due to the low expression of lncRNAs and the computational complexity of experimental data analysis. Although some computational tools have been proposed to predict circRNAs, their performance still needs to be improved.

In this study, we proposed StackCirRNAPred for classifying circRNAs from other lncRNAs using ensemble learning ideas based on the stacking strategy to fuse multiple single classifiers. StackCirRNAPred outperforms other methods on human and mouse datasets. The performance of StackCirRNAPred is also the best when compared to a single classifier. This shows that the fusion of multiple classifiers can better integrate features from different sources, and is a means to improve the sensitivity and specificity of the model.

Even though StackCirRNAPred still achieved better performance on the mouse dataset, this does not prove the applicability of our method to other species, as a large number of experiments or new modifications to the method are needed, which will be the focus of our future work. To our knowledge, there is currently no method for cross-species circRNA prediction. With the development of biotechnology and circRNA research becoming more advanced, it is believed that high-quality circRNAs of more and more species will be discovered and annotated, and that a large enough set of high-quality data will be available to support future research on cross-species circRNA prediction methods.

## Conclusion

Since there is still much room for improvement in the computational classification of circRNAs from other lncRNAs, we proposed StackCirRNAPred based on the stacking strategy to distinguish circRNAs from other lncRNAs. Our method showed good predictive performance on both human and mouse datasets, and the prediction performance was significantly better than other methods. It demonstrated the effectiveness and robustness of our method. We hope that StackCirRNAPred can complement existing circRNA identification methods and contribute to down-stream research.

## Supplementary Information

**Additional file 1**: The datasets used in this paper: human datasets and mouse datasets.

**Additional file 2**: **Table S1**. The Optimal Feature Set of 110-dimensional.

## Data Availability

All data generated or analyzed during this study are included in this published article and its additional information files. Human (GRCh37) and mouse (NCBI37) reference genome files can be downloaded from UCSC Genome Browser (https://hgdownload.soe.ucsc.edu/downloads.html). Human lncRNAs data can be downloaded from GENCODE (GRCh37) (https://www.gencodegenes.org/human/release_40lift37.html). The mouse circRNA annotation bed file can be downloaded from circBase (http://circbase.org/cgi-bin/downloads.cgi). The mouse lncRNAs data can be downloaded from GENCODE (Release M1) (https://www.gencodegenes.org/mouse/release_M1.html). The codes in this study are available at https://github.com/xwang1427/StackCirRNAPred.
